# People Counting by Dense WiFi MIMO Networks: Channel Features and Machine Learning Algorithms †

**DOI:** 10.3390/s19163450

**Published:** 2019-08-07

**Authors:** Sanaz Kianoush, Stefano Savazzi, Vittorio Rampa, Monica Nicoli

**Affiliations:** 1 National Research Council of Italy (CNR), Institute of Electronics, Computer and Telecommunication Engineering (IEIIT), Piazza Leonardo da Vinci 32, 20133 Milano, Italy; 2 Politecnico di Milano, Department of Management, Economics and Industrial Engineering (DIG), Piazza Leonardo da Vinci 32, 20133 Milano, Italy

**Keywords:** crowd sensing, MIMO WiFi, machine learning, 5G, cloud computing

## Abstract

Subject counting systems are extensively used in ambient intelligence applications, such as smart home, smart building and smart retail scenarios. In this paper, we investigate the problem of transforming an unmodified WiFi radio infrastructure into a flexible sensing system for passive subject counting. We first introduce the multi-dimensional channel features that capture the subject presence. Then, we compare Bayesian and neural network based machine learning tools specialized for subject discrimination and counting. Ensemble classification is used to leverage space-frequency diversity and combine learning tools trained with different channel features. A combination of multiple models is shown to improve the counting accuracy. System design is based on a dense network of WiFi devices equipped with multiple antennas. Experimental validation is conducted in an indoor space featuring up to five moving people. Real-time computing and practical solutions for cloud migration are also considered. The proposed approach for passive counting gives detection results with 99% average accuracy.

## 1. Introduction

Internet of Things (IoT) and new ubiquitous connectivity paradigms beyond 5G have created unprecedented dynamics for opportunistic sensing by exploiting low-cost radio devices [1,2]. Such new sensing modalities leverage the cross-fertilization of computing and communication technologies and are typically achieved through the transformation of natural, stray or ambient radio frequency (RF) radiation into new sensing ways [3] to probe the environment and the people moving inside it. In particular, the use of devices with built-in radio modems (e.g., WiFi or cellular-enabled devices) and Multiple–Input Multiple–Output (MIMO) capabilities, as envisioned by 5G for device-free (or passive) body motion sensing [1], is becoming attractive in many fields, such as smart homes and assisted living applications. Device-free radio sensing provides privacy-preserving monitoring with increased robustness to environmental conditions with respect to video-based systems [4,5], such as in case of fire or variable light conditions.

**Figure 1:**
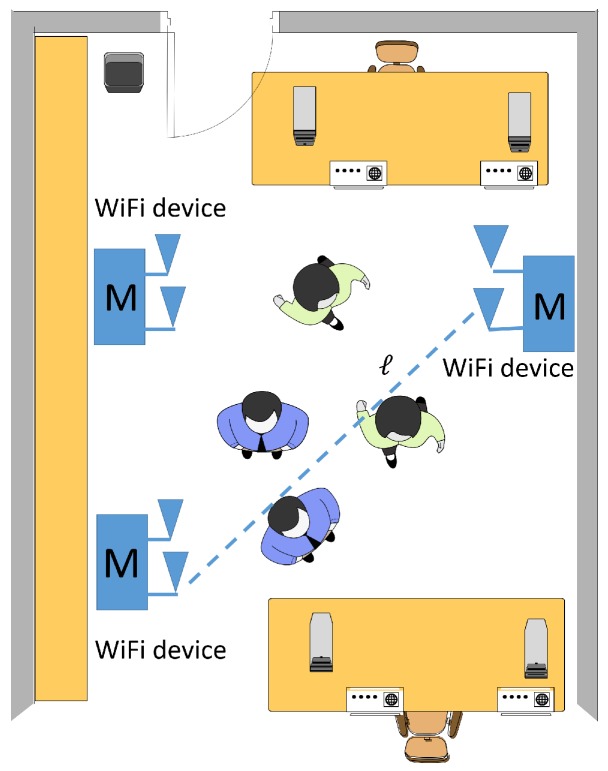
Crowd sensing using a WiFi network composed by $N_{t}$ transmitting and $N_{r}$ receiving MIMO WiFi devices. Each MIMO WiFi device is equipped with *M* antennas while $M_{t}=N_{t}M$ and $M_{r}=N_{r}M$ are the total number of transmitting and receiving antennas, respectively.

In this paper, we address the specific problem of passive people counting in an indoor space covered by a network of MIMO WiFi devices. The proposed counting system tracks and classifies the perturbations of the electromagnetic field maintained by a WiFi network to detect and discriminate multiple subjects (namely the targets) that cause such perturbations. As depicted in the scenario of Figure 1, target detection and counting is based on real-time processing of the radio channel that is measured by a dense network of MIMO WiFi devices configured as transmitters (TXs) and receivers (RXs) according to the specific wireless protocol.

### Contributions

Capturing crowd dynamics (i.e., subject counting and tracking) by signal analytics in IoT networks is an emerging topic in research as well as in practical implementation of crowd management systems for smart city applications [6]. By using video footage, computer vision approaches allow accurate crowd monitoring. However, deploying a camera network is costly and inhibits scalability for events that happen infrequently. In addition, video systems have often different specific installation requirements and privacy constraints, as well. On the other hand, supported by a large installation basis, currently available IoT devices offer an excellent instrumentation ground for crowd monitoring, thanks to the multitude of available sensors and radio interfaces (e.g., Bluetooth, WiFi, Thread and ZigBee). Passive crowd monitoring by wireless networks is a new emerging topic of research but some first experimental works can be found in the literature. In reference [7], the authors proposed an algorithm to localize and count multiple targets. In order to address the non-linearity of the impact of multiple subjects on the radio signals, they proposed a successive cancellation algorithm to iteratively determine the number of subjects by modelling the indoor human trajectories as a state transition process, using an indoor human mobility model and integrating all information into a Conditional Random Field (CRF) to simultaneously localize and count the subjects. In reference [8], the authors proposed a non-image people counting system based on a Deep Neural Network (DNN) model using fine-grained physical-layer wireless signatures such as WiFi CSI (Channel State Information) data. Real test-bed experiments showed that the proposed system can achieve an average correct classification rate up to 88% when estimating the crowd size in indoor scenarios with up to nine people. In reference [9], the authors suggested a people counting algorithm using Impulse Radio Ultra-WideBand (IR-UWB) radar sensors, equipped with antennas which have narrow beam width. The system performances have been validated in a representative environment, showing results with large accuracy. However, the use of dedicated UWB radar sensors is still an expensive choice for applications in massive IoT smart spaces, where thousands of IoT devices are deployed.

This paper proposes the transformation of a dense MIMO WiFi network into a passive crowd sensing system by exploiting Machine Learning (ML) tools that process multi-dimensional CSI extracted from different PHY (PHYsical layer) frames, MIMO antennas and sub-carriers. Dense MIMO networks pose remarkable scalability challenges in signal processing due to the increase of the dimensions of the CSI data that are usually represented as tensors or multi-dimensional arrays. Therefore, unlike previous approaches, we propose here ML tools that are designed to fully exploit the statistical coherence of the CSI data over this multi-dimensional space domain (i.e., antenna, frequency, time) in order to extract compact but maximally informative CSI features. In other words, CSI data tensors are pre-processed to produce custom-designed features that are organized in accordance to the meaning of the underlying data dimensionality.

Based on such premise, ensemble classification and ML approaches based on different multi-dimensional features are compared, such as Bayesian techniques, recurrent Long-Short Term Memory (LSTM) neural network and Feed-Forward Neural Network (FF-NN) models [10]. Finally, considering the problem of scalability to large monitored areas (e.g., system deployed in buildings or multiple rooms), we consider the problem of transferring part of the data analytics to an edge-cloud system to support real-time monitoring [11].

The paper is organized as follows: Section 2 discusses the selection of multi-dimensional radio channel features according to the space-frequency MIMO configurations. Section 3 presents the computing architecture including ML tools and the edge-cloud computation system, while Section 4 describes the experimental validation setup and presents some numerical results. Finally, some conclusions are drawn in Section 5.

## 2. CSI Features for Subject Counting

We consider the problem of extracting information about the number $C\in \left\{0,1,\ldots ,N\right\}\subset N$ of human targets moving in a monitored area X by processing CSI data measured over a broadband multi-carrier MIMO network deployed in the area. As illustrated in Figure 1, the network is composed of $N_{t}$ transmitting and $N_{r}$ receiving devices, each equipped with *M* antennas. Modulation is based on Orthogonal Frequency-Division Multiplexing (OFDM). The overall network MIMO system is thus characterized by $M_{t}=N_{t}M$ transmitting and $M_{r}=N_{r}M$ receiving antennas. Any target moving inside the area modifies the radio propagation between the devices and thus is expected to affect the MIMO CSI in terms of mean power (due to body induced attenuation), fading (due to body movements) and space-frequency selectivity (due to the alteration of the multipath dispersion).

We propose to observe the baseband channel response over the space-frequency-time domains to capture the overall impact and extract all available information about the presence and number of targets in the area. We assume that the MIMO channel can be estimated at the receiver side at the discrete time instants t∈T={1,2,…,T}, spanning over *T* OFDM symbols (or frames). These observations are expected to embed a characteristic footprint of the channel variations induced by people movements in the monitored area.

### 2.1. MIMO-OFDM Channel Response and CSI Data Sets

Using a conventional channel estimation method based on standard-defined pilot sub-carrier arrangement [12], the MIMO channel response is monitored in the frequency domain over *K* pilot sub-carriers $f\in F=\{f_{1},f_{2},\ldots ,f_{K}\}$. OFDM training symbols for channel estimation are usually multiplexed with information symbols in each data frame according to the adopted WiFi standard. The complex baseband channel response observed on frequency f∈F, over the link $\ell =\ell (a_{t},a_{r})$ between the transmitting antenna $a_{t}=1,\ldots ,M_{t}$ and receiving antenna $a_{r}=1,\ldots ,M_{r}$, at time t∈T, is denoted as $H_{f,\ell ,t}$, where the index ℓ∈L={1,2,…,L} ranges over the $L=M_{t}M_{r}$ radio links.

Focusing on subject counting, we highlight the channel response in the space and frequency domains as the input for feature processing. In particular, we define the L×1 space-domain channel response for the sub-carrier *f* as

(1) $$H_{t}^{f}={\left[H_{f,\ell ,t}\right]}_{\ell \in L}\;,$$

while the K×1 frequency-domain response for the link *ℓ* is defined as

(2) $$H_{t}^{\ell }={\left[H_{f,\ell ,t}\right]}_{f\in F}\;.$$

Finally, we define the overall space-frequency CSI as $H_{t}={\left[H_{f,\ell ,t}\right]}_{f\in F,\ell \in L}$. These CSI sets are expected to embed information about the number of targets *C* and are thus used in the next sections to extract the features for target counting.

### 2.2. Space-Frequency Domain CSI Features

In what follows, features are built according to the CSI measured over the space (1) and the frequency (2) domains. The CSI strength observed at time t∈T on sub-carrier f∈F and link ℓ∈L, is defined in dB scale as $s_{f,\ell ,t}={\left|H_{f,\ell ,t}\right|}_{\mathrm{dB}}^{2}$. Relevant features can be extracted from these CSI data for counting and crowd size classification. In particular, body movements are expected to modify the statistical properties of the CSI in both the space and frequency domains, such as the strength moments evaluated as average over time in a given space-frequency sample (e.g., the mean and variance, related to the body induced attenuation and fading), as well as the correlation over the sub-carrier frequencies (accounting for the crowd impact on the multipath power-delay profile) and over the antennas (accounting for the impact on the MIMO power-space profile). Thereby, in the following, we consider the statistics of the overall CSI power space-frequency profile. The frequency-domain CSI strength vector for link *ℓ* at time *t* is defined as

(3) $$s_{\ell ,t}={\left[s_{f_{1},\ell ,t}\cdots s_{f_{K},\ell ,t}\right]}^{T}$$

which collects the measurements of the channel response over all the sub-carriers for the link ℓ∈L and, similarly, the space-domain CSI vector

(4) $$s_{f,t}={\left[s_{f,1,t}\cdots s_{f,L,t}\right]}^{T}$$

collecting the channel measurements over all active MIMO links, for the pilot sub-carrier f∈F.

Considering a single channel strength sample $s_{f,\ell ,t}$ extracted from the frequency or space domain vectors, we choose four distinctive statistical indicators that provide a representation of the corresponding probability function $s_{f,\ell ,t}∼\mathrm{Pr}\left[s_{f,\ell ,t}\right]$, namely

(5) $$\begin{array}{c} \mu_{f,\ell }=E_{t}\left[s_{f,\ell ,t}\right]\;,\\ \sigma_{f,\ell }=\sqrt{E_{t}\left[{\left(s_{f,\ell ,t}-\mu_{f,\ell }\right)}^{2}\right]}\;,\\ \zeta_{f,\ell }=E_{t}\left[{\left(\frac{s_{f,\ell ,t}-\mu_{f,\ell }}{\sigma_{f,\ell }}\right)}^{3}\right]\;,\\ \kappa_{f,\ell }=E_{t}\left[{\left(\frac{s_{f,\ell ,t}-\mu_{f,\ell }}{\sigma_{f,\ell }}\right)}^{4}\right]\;. \end{array}$$

where $\mu_{f,\ell }$ and $\sigma_{f,\ell }$ are the mean and the standard deviation, while $\zeta_{f,\ell }$ and $\kappa_{f,\ell }$ are the skewness (i.e., third moment) and kurtosis (i.e., fourth moment) coefficients herein evaluated over consecutive time samples (i.e., WiFi frames). In particular, the skewness indicator captures the distance between mean and mode values, while kurtosis measures the concentration of the probability mass in the tails of the distribution.

**Figure 2:**
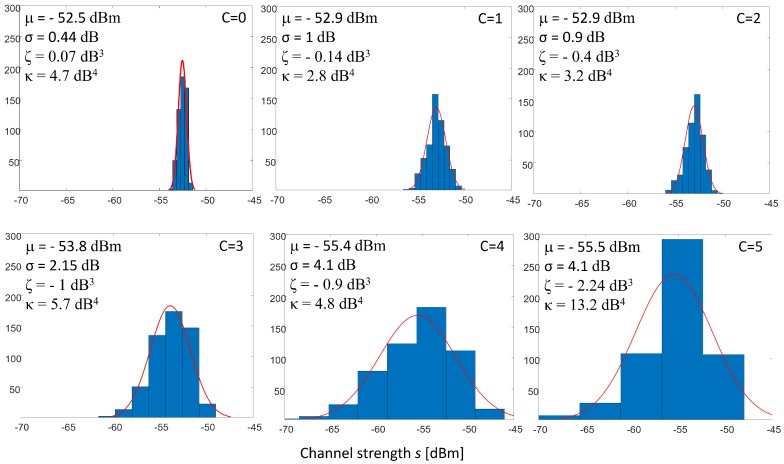
Histogram $p_{f,\ell }\left(s\right)$ of the CSI strength sample $s_{5,5}$ in dBm, for $f=f_{5}$ and ℓ=5, and the corresponding extracted features including mean [dBm], standard deviation [dB], skewness [dB${}^{3}$], and kurtosis [dB${}^{4}$] over time series. Measurements are obtained from the proposed system deployed in the layout shown in Figure 3 in the empty space case (C=0) and for the subject number *C* ranging from 1 up to 5.

Both skewness and kurtosis features are affected by the number of targets *C* as illustrated in Figure 2. The figure shows the empirical distribution of the CSI strength measurements $p_{f,\ell }\left(s\right)=\mathrm{Pr}\left[s_{f,\ell ,t}\right]$ built by observing the statistics over a number *T* of consecutive MIMO WiFi frames *t*, for the link index 5 (ℓ=5) and the sub-carrier index 5 ($f=f_{5}$). Corresponding features include the mean μ, the standard deviation σ, the skewness ζ and the kurtosis κ. As shown in the figure, the values of the CSI indicators change with the number of the subjects inside the area. For example, mean, standard deviation, kurtosis and skewness values are -52.9 dBm, 1 dB, -0.14 dB${}^{3}$, and 2.8 dB${}^{4}$ for C=1 and -55.5 dBm, 4.1 dB, -2.24 dB${}^{3}$, and 13.2 dB${}^{4}$ for C=5, respectively.

**Figure 3:**
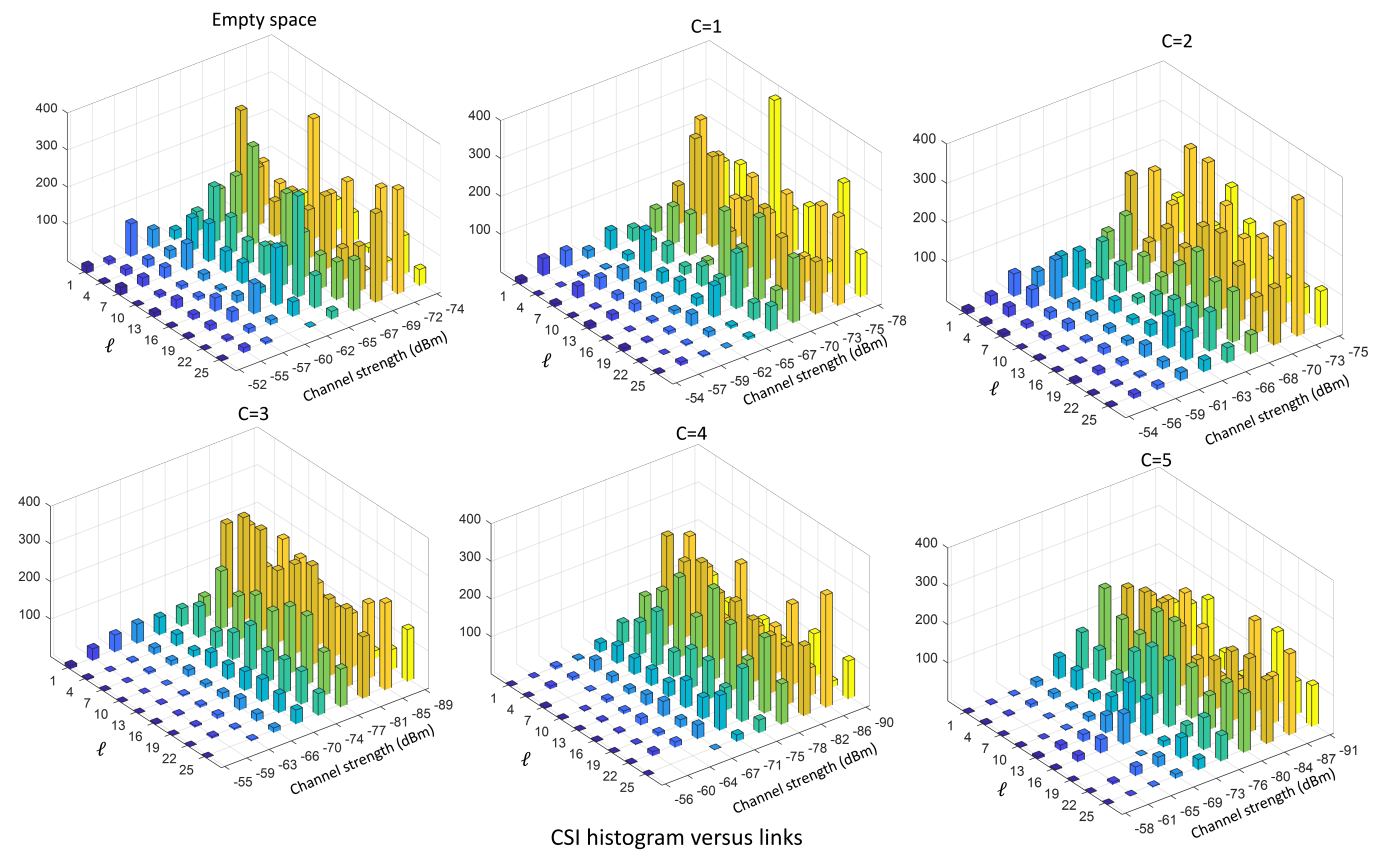
CSI histograms versus links $p_{\ell }\left(s\right)$ with ℓ=1,…,27, for the number of people ranging from C=0 (empty environment) up to C=5 evaluated by aggregating data over K=30 sub-carriers.

**Figure 4:**
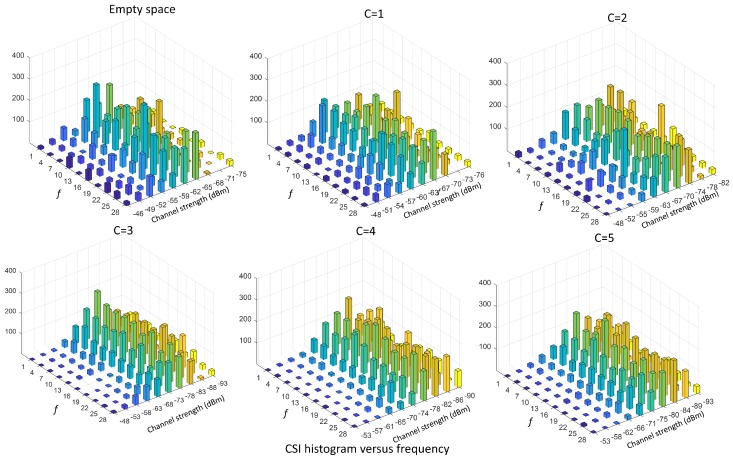
CSI histograms versus frequency $p_{f}\left(s\right)$ with $f=f_{1},\ldots ,f_{30}$, for the number of people ranging from C=0 (empty environment) to C=5 evaluated by aggregating data over L=27 links.

A feature embodying the moments of all orders is the CSI distribution. Figure 3 and Figure 4 show the CSI distribution versus links $p_{\ell }\left(s\right)=\mathrm{Pr}\left[s_{f,\ell ,t}=s|\ell \right]$, for ℓ∈L, and versus frequency $p_{f}\left(s\right)=\mathrm{Pr}\left[s_{f,\ell ,t}=s|f\right]$, for f∈F, obtained in the form of histogram from real measurements taken in an indoor environment, with number of targets ranging from C=0 (i.e., empty environment) up to C=N=5. In particular, Figure 3 shows the CSI distribution $p_{\ell }\left(s\right)$ versus link *ℓ* built by aggregating the samples over the frequency domain, while Figure 4 represents the distribution $p_{f}\left(s\right)$ versus sub-carrier *f* by aggregating over the links. As it can be seen from these figures, the CSI distributions in both space and frequency domains are sensitive to the number of targets. Statistics change more significantly for 0≤C≤2, whereas, for 2<C≤5, the dependence due to the number of targets is less evident but still present and can be fully exploited by using the CSI distribution to build these features.

In what follows, real-time subject counting is based on the analysis of time series of *T* consecutive MIMO WiFi frames. The four statistical indicators in (5) are thus evaluated over both frequency and space domains, resorting to temporal sequences, or features, of *T* samples or frames. Notice that, as highlighted in Section 3 and Section 4, the number *T* of frames per feature depends on the learning model. For the frequency domain, we define four time series vectors

(6) $$\begin{array}{c} \mu_{\ell }={\left[\mu_{1,\ell }\cdots \mu_{T,\ell }\right]}^{T}\;,\\ \sigma_{\ell }={\left[\sigma_{1,\ell }\cdots \sigma_{T,\ell }\right]}^{T}\;,\\ \zeta_{\ell }={\left[\zeta_{1,\ell }\cdots \zeta_{T,\ell }\right]}^{T}\;,\\ \kappa_{\ell }={\left[\kappa_{1,\ell }\cdots \kappa_{T,\ell }\right]}^{T}\;. \end{array}$$

where $\mu_{t,\ell }$ is defined as $\mu_{t,\ell }=E_{f}\left[s_{f,\ell ,t}\right]$ while $\sigma_{t,\ell }$, $\zeta_{t,\ell }$, and $\kappa_{t,\ell }$ are defined similarly as in (5) but replacing time index with the frequency one while expectations are made over f∈F. For space domain, the corresponding features are described by the vectors

(7) $$\begin{array}{c} \mu_{f}={\left[\mu_{1,f}\cdots \mu_{T,f}\right]}^{T}\;,\\ \sigma_{f}={\left[\sigma_{1,f}\cdots \sigma_{T,f}\right]}^{T}\;,\\ \zeta_{f}={\left[\zeta_{1,f}\cdots \zeta_{T,f}\right]}^{T}\;,\\ \kappa_{f}={\left[\kappa_{1,f}\cdots \kappa_{T,f}\right]}^{T}\;. \end{array}$$

where $\mu_{t,f}$ is defined as $\mu_{t,f}=E_{\ell }\left[s_{f,\ell ,t}\right]$ while $\mu_{t,f}$, $\sigma_{t,f}$, $\zeta_{t,f}$, and $\kappa_{t,f}$ are defined as in (5), by replacing the time index with the link one while expectations are made over ℓ∈L. Features (6) and (7) can be also represented as the sets ${\left[\mu_{t,\ell },\sigma_{t,\ell },\zeta_{t,\ell },\kappa_{t,\ell }\right]}_{t=1}^{T}$ and ${\left[\mu_{t,f},\sigma_{t,f},\zeta_{t,f},\kappa_{t,f}\right]}_{t=1}^{T}$, respectively. Both feature sets (6) and (7) are used an input to machine learning tools detailed in Section 3 and Section 4.

**Figure 5:**
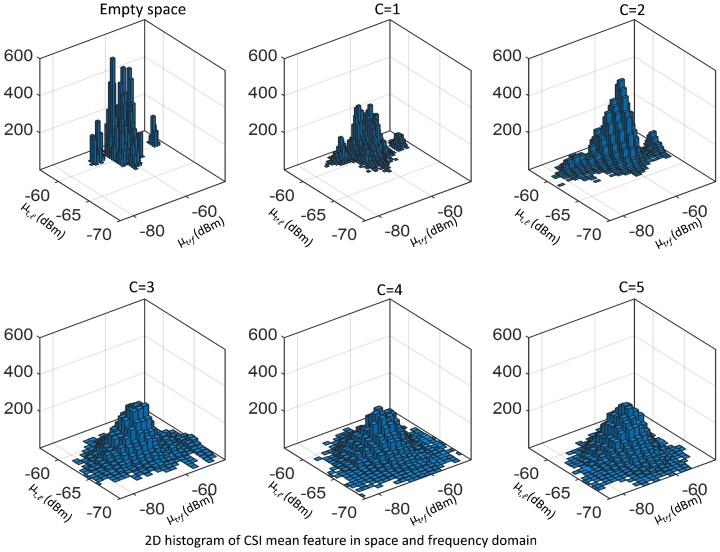
2D histogram of CSI mean in space (i.e., $\mu_{t,f})$ and frequency (i.e., $\mu_{t,\ell })$ domain for the empty space case and the subject number ranging from 1 up to 5.

As an example, Figure 5 shows the 2D histograms of the CSI mean feature $\mu_{t,f}$ and $\mu_{t,\ell }$ in the space and frequency domains as well, for varying number *C* of people in the range 0≤C≤5. As shown in the figure, the distribution properties are substantially affected by the number of subjects. Again, the sensitivity is higher for *C* in the range 0≤C≤2. Similar effects (not shown here) are observed considering the deviations, skewness and kurtosis in (6) and (7).

The impact of body movements on higher order CSI moments over multiple antennas (space domain) and sub-carriers (frequency domain) is still unexplored in the literature. In the following sections, we deepen the topic by proving that, compared to the unobstructed environment, the presence of the target(s) affects both the space- and frequency-domain CSI moments and such alterations can be exploited as reliable features for target counting.

## 3. Computing Architecture and Processing Tools for Subject Counting

**Figure 6:**
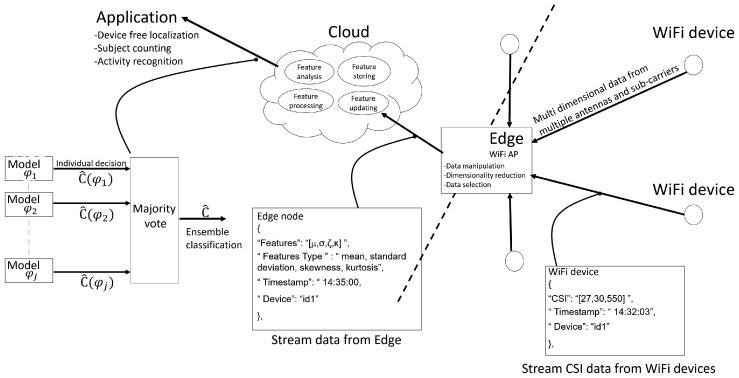
Edge-cloud computing architecture for CSI data manipulation, feature processing and subject counting.

In this section, we highlight the computing architecture and the CSI processing tools that are implemented for real-time subject counting. The proposed system relies on computing and caching stages (see Figure 6) that run inside the WiFi Access Point (AP) device, serving as edge node and inside a computing device, deployed remotely that serves as cloud node. The WiFi AP is in charge of the segmentation and extraction of the CSI features (Section 2) obtained from the CSI measurements performed by the deployed WiFi devices. The cloud node processes the features received from the WiFi AP to infer the number of subjects and, possibly, forwards this information via the MQTT (Message Queuing Telemetry Transport) protocol [13] to different subscribers or applications [14]. As depicted in the same figure, the WiFi AP, serving as edge node, exposes a set of REpresentational State Transfer (REST) Application Programming Interfaces (APIs) that are used by the WiFi devices to send the CSI data (via HTTP POSTs) encoded in a Java Script Object Notation (JSON) format [15]. The AP node extracts the CSI features used for subject counting (Section 2) and publishes such features via the MQTT protocol. Finally, the cloud device, acting as MQTT subscriber, processes such features for inference and, in turn, publishes the detected targets results.

In what follows, we discuss the ML tools for CSI feature processing that are implemented in the cloud to perform subject counting.

### 3.1. Machine Learning Tools for CSI Feature Processing

**Figure 7:**
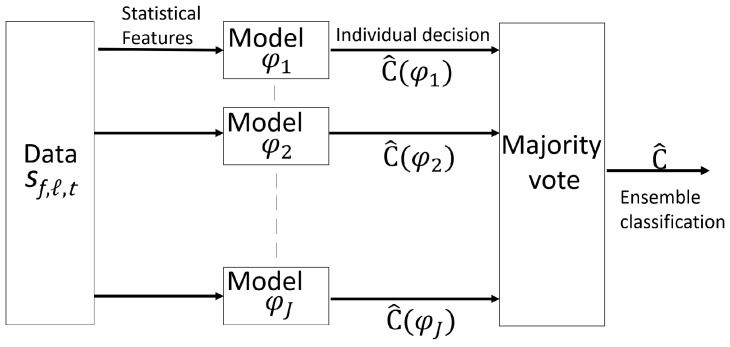
Block diagram of the ensemble classifier where the input data are the CSI samples. Multiple features, that are used as input to the model blocks, include mean μ, standard deviation σ, skewness ζ, and kurtosis κ computed in the frequency $\left\{\mu_{\ell },\sigma_{\ell },\zeta_{\ell },\kappa_{\ell }\right\}$ (6) and space $\left\{\mu_{f},\sigma_{f},\zeta_{f},\kappa_{f}\right\}$ (7) domains. LSTM and FF-NN models are used in model blocks $\varphi_{j}$ and an individual decision is made by each block. Ensemble classification $\hat{C}$ is based on majority vote of individual decisions $\hat{C}\left(\varphi_{j}\right)$.

ML tools [16] are here adopted for real-time processing of the CSI features extracted as in Section 2 from multidimensional channel data observed over time, frequency and links to provide an estimate $\hat{C}$ of the number of subjects *C* moving in the monitored area. The learning process benefits from the combination of multiple models that are individually trained and optimized based on the input time series of CSI features defined in (6) and (7) in both frequency and space domain. More specifically, as highlighted in Figure 7, we construct ensembles that independently train *J* individual Neural Network (NN) models $\varphi_{j}$ with j=1,…,J. Ensemble approaches for learning have two major components, that is, a method for creating individual NNs and a method for combining NNs, typically by weighted or unweighted, voting [17]. We consider two NN types, namely the LSTM and the FF-NN models. Each network is optimized, by supervised learning, for a specific feature vector. The input for each model $\varphi_{j}$ thus consists of space-frequency domain features (6) and (7) obtained in a specific time frame. Ensemble methods combine multiple learning models $\varphi_{j}$ with the goal of improving the robustness over a single estimator [18]. As highlighted in Section 4, considering the four CSI feature vectors corresponding to the first four statistical moments of the input CSI data shown in Section 2, the proposed ensemble model exploits the output of *J* classification algorithms. Popular combining architectures for constructing ensemble are the bagging [17] and boosting [19] algorithms. Here, a bagging approach is adopted while unweighted major voting is chosen to combine the outputs $\hat{C}\left(\varphi_{j}\right)$ of the individual NN classifiers to update the new detection estimate. In Section 4, optimal combinations of NNs to maximize the system performances are investigated as well.

### 3.2. Kullback-Leibler Divergence for Processing of CSI Distribution Features

The Kullback-Leibler (KL) divergence [20] is commonly used to measure the similarity between two distributions. This metric is here employed to design a further tool for subject counting (in addition to ML tools proposed in the previous section), based on the evaluation of the similarity between the CSI distributions built from the training phase and the input CSI observations. We define as $q_{f,\ell }\left(s|C\right)=\mathrm{Pr}\left[s_{f,\ell ,t}=s|C\right]$ the CSI distribution obtained from training on sub-carrier *f* and link *ℓ* (see Section 4), when *C* subjects are moving in the space. For C=1,…,N targets, the subject counting process consists in computing *N* KL divergence measures (or likelihoods, in the Gaussian case) between the observed input distribution $p_{f,\ell }\left(s\right)=\mathrm{Pr}\left[s_{f,\ell ,t}=s\right]$ and the *N* distributions $q_{f,\ell }\left(s|C\right)$ collected at training stage for C=1,…,N. In particular, the KL divergence for *C* target(s) is defined as

(8) $${\mathrm{DKL}}_{f,\ell }\left(C\right)=D_{KL}(p_{f,\ell }\left(s\right)||q_{f,\ell }\left(s|C\right))=\sum_{s}p_{f,\ell }\left(s\right)\log\frac{p_{f,\ell }\left(s\right)}{q_{f,\ell }\left(s|C\right)}$$

Divergence (8) can be used to define feature vectors, similarly to what done for mean, standard deviation, skewness and kurtosis, that are used as input to learning tools (Section 4). In particular, considering processing in the frequency domain, the minimum divergence estimator of the number of targets $\hat{C}$ can be written as

(9) $${\hat{C}}_{\ell }=\underset{C=1,\ldots ,N}{\mathrm{argmin}}\sum_{k=1}^{K}{\mathrm{DKL}}_{f_{k},\ell }\left(C\right)$$

and, similarly, in the space domain, it is

(10) $${\hat{C}}_{f}=\underset{C=1,\ldots ,N}{\mathrm{argmin}}\sum_{\ell =1}^{L}{\mathrm{DKL}}_{f,\ell }\left(C\right).$$

For both cases, the final decision is based on majority voting on $C_{\ell }$, in frequency and $C_{f}$, in space domain, respectively.

## 4. Subject Counting: Results and Discussions

**Figure 8:**
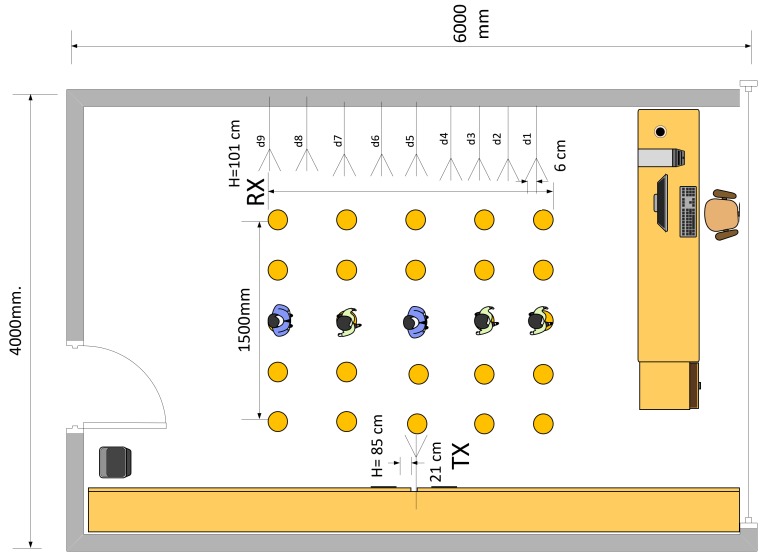
Deployment layout for the experimental subject counting system including $N_{t}=1$ TX and $N_{r}=9$ RX devices with M=3 antennas for each device. Distance between antennas at receiver is 6 cm, while transmitter antennas are spaced apart by 21 cm. The monitored area size is 6×4 m${}^{2}$. Up to N=5 people enter this area and walk along the middle line thus blocking the LOS path between TX and RXs.

The experimental activities have been conducted inside the indoor lab environment shown in Figure 8. We employed a network of 10 MIMO WiFi devices that have been distributed over a monitored area having size 6 × 4 $m^{2}$. A single MIMO WiFi device (i.e., $N_{t}=1$) is acting as TX while $N_{r}=9$ MIMO WiFi devices are configured as RXs; each MIMO WiFi device is equipped with M=3 antennas. Devices are deployed in the monitored area to capture the target presence at different positions. Usually, the sensing network is not regularly deployed around the perimeter of the monitored area (as also proposed in the experimental setup); however, clustering of nodes might affect the performance, as analyzed in reference [21] for localization purposes. Although not explicitly addressed in this paper, node layout can be optimized during a pre-deployment stage by using, for example, a Cramer-Rao bound based analysis to evaluate the impact of the geometric factor on the inference system performance [21]. This experimental section instead focuses on system validation in a real environment where the deployment of the sensors is assigned and node optimization is not allowed. Deployment optimization to improve the counting accuracy is however an interesting and open problem, also considering that passive detection in large buildings is typically obtained by scaling up the number of MIMO WiFi devices (for dense deployments). In the proposed tests, all MIMO WiFi devices are configured in monitor mode and working in the 5.32 GHz band (i.e., WiFi band 2, channel 64, OFDM symbol sub-carrier spacing 312.5 kHz and nominal bandwidth equal to 20 MHz). The monitor mode allows the receivers to observe the CSI values on the considered channel without explicit IP handshaking procedures. The TX device is programmed to inject (i.e., transmit) custom IEEE 802.11n PHY Protocol Data Units (PPDU) structured as standard High-Throughput (HT) greenfield WiFi format [22] including preamble, MAC addresses, header and payload: injected frames are sent at regular time intervals of 10 ms. In our tests, the TX device acts as an access point while the RX devices are collecting and measuring CSI reports. Modified chipset firmware and kernel [23] have been used to obtain the CSI samples of received IEEE 802.11n data frames. The adopted chipset is the Intel Wireless Link 5300 working as a MIMO-OFDM baseband modem. The modified driver allows to extract the standard CSI reports for uni-cast/broadcast packets. CSI reports are organized into a JSON format as described in Figure 6. Each JSON object tracks the CSI data set, organized by frequency, antenna and time frames, the device identifiers and the timestamps.

As shown in Figure 8, the MIMO WiFi devices are arranged to cover the whole monitored area and to capture signals obstructed by moving objects. The overall number of TX and RX antennas is $M_{t}=3$ and $M_{r}=27$, respectively. CSI data are collected over K=30 sub-carriers. Up to N=5 people are moving inside the room across the LOS (Line Of Sight) as shown in Figure 8. The LOS path indicates the link path, where TX and RX are facing in straight line.

### 4.1. Classifier tools for Subject Counting

In our experimental tests, the crowd size increases from C=1 up to C=5 people while body motion patterns are performed inside the monitored area as shown in Figure 8. In particular, in the proposed set-up, 1≤C≤5 people enter the area one by one, staying/wandering for 10 s across the LOS path and then leaving sequentially one by one. Thus, up to N=5 people may occupy simultaneously the monitored area. To detect the presence and estimate the number of people, we exploit state-of-the-art classifiers that employ the first two statistical channel strength moments as CSI features, namely the mean and standard deviations. In particular, we compare the performance of FF-NN, LSTM and maximum likelihood approaches [24] implemented separately, as routinely done in device-free localization (DFL) applications. We evaluate the performance in terms of accuracy, defined as the probability of correct classification. In addition, we assess also the performance of the KL divergence minimization approach (Section 3.2) that can be interpreted as a generalization of the maximum likelihood techniques, under non-Gaussian assumptions.

Table 1 shows the accuracy results for the maximum likelihood approach. The CSI distribution $p_{f,\ell }\left(s\right)$ is approximated here as normal with mean and standard deviation terms altered by the presence of multiple targets. Numerical results show that despite the system can accurately discriminate between C=1 and C=2 targets, when the crowd size increases the counting accuracy performance drops to 0.55 and 0.30, respectively.

In Table 2, we assess the performance of the subject counting system using the KL divergence minimization. In particular, during the training phase, we collect CSI measurements from L=27 links and K=30 sub-carriers and extract the corresponding distributions $q_{f,\ell }\left(s|C\right)$ for varying number of targets C=1,…,5. Real-time processing is then implemented separately in the space (10) and frequency domain (9) by choosing the minimum divergence with respect to the observed (input) distribution $p_{f,\ell }\left(s\right)$. Table 2 summarizes the accuracy performance obtained in the space and frequency domain processing, respectively. As seen previously according to the maximum likelihood approach, we observe a significant performance degradation when the number of targets increases.

**Figure 9:**
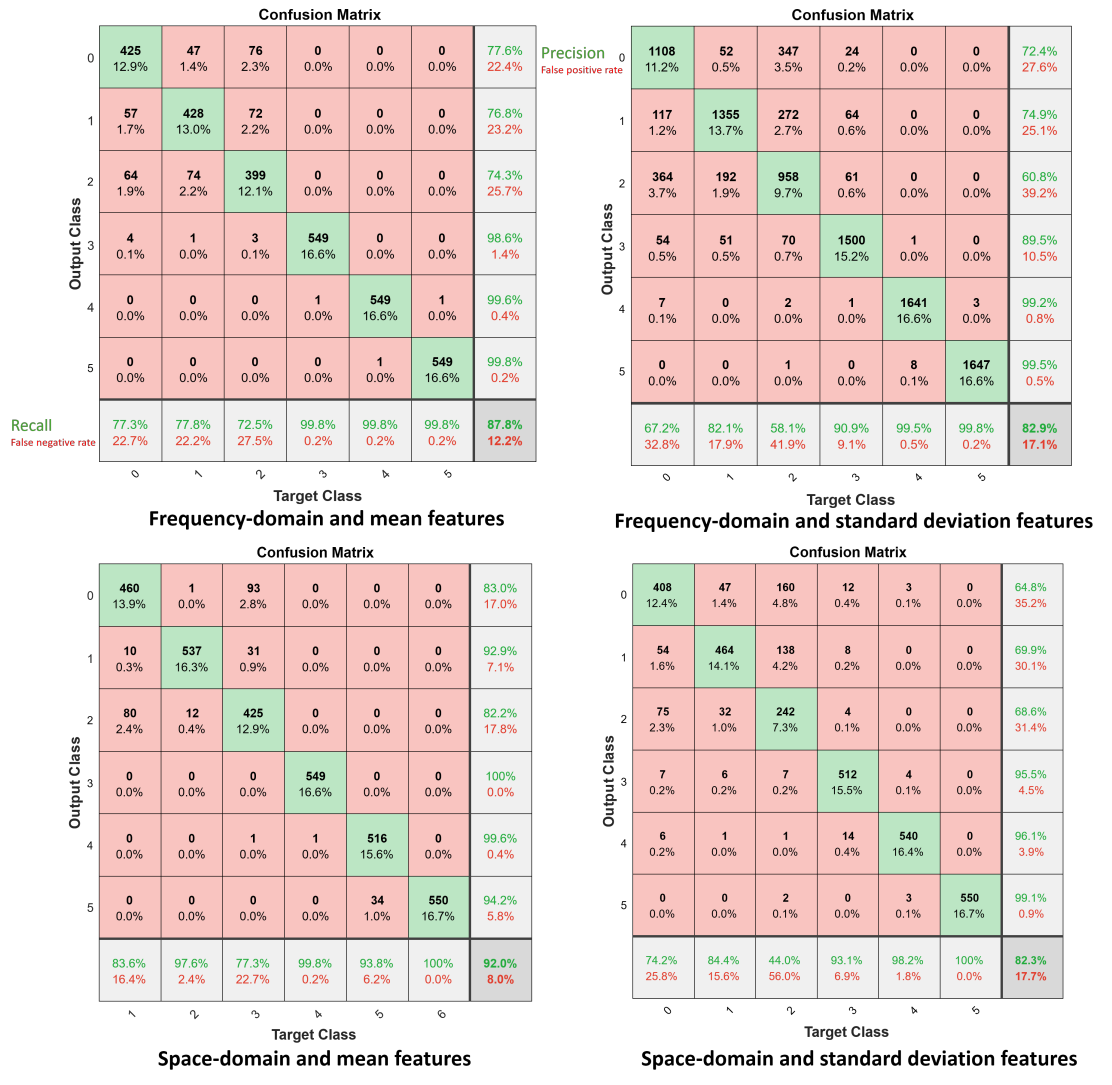
Confusion matrices for the FF-NN classifier for subject counting, C=0,1,2,...,5 where C=0 corresponds to the empty environment. Top figure: frequency domain CSI features: mean (**left**) and standard deviation (**right**). Bottom figure: space domain CSI features: mean (**left**) and standard deviation (**right**).

**Figure 10:**
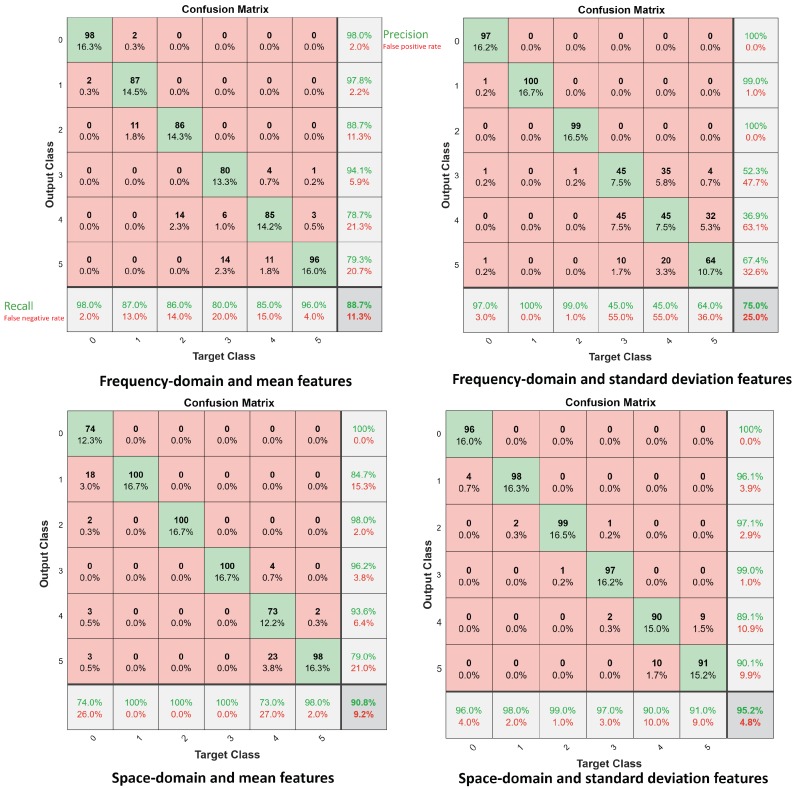
Confusion matrices for the LSTM classifier for subject counting, C=0,1,2,...,5. Top figures: frequency domain CSI features: mean (**left**) and standard deviation (**right**). Bottom figures: space domain CSI features: mean (**left**) and standard deviation **(right**).

Figure 9 and Figure 10 represent the confusion matrix results using the mean and standard deviation CSI features, in the space-frequency domain, when FF-NN (Figure 9) and LSTM (Figure 10) networks are chosen as classifiers, respectively. Confusion matrices highlight the average accuracy, as well as the precision and recall figures for varying number of targets 0≤C≤5. Compared with the previous techniques, the average accuracy improves. Moreover, space domain CSI features (7) provide some advantages compared with frequency domain terms. In the following section, an ensemble classifier is adopted to combine multiple CSI features. In addition, we assess also the inclusion of skewness and kurtosis metrics (5) to further improve the system performances.

### 4.2. Ensemble Learning and Bagging: Channel Features and Comparative Analysis

In this section, we assess the performances of an ensemble method (Figure 7) to combine different models with the goal of improving the robustness over the single estimators highlighted in the previous sections. In particular, the proposed ensemble model exploits the output of different classification algorithms, separately trained, that use four different CSI features as inputs, namely the mean, standard deviation, skewness and kurtosis (5). Table 3 shows the accuracy results for the same layout of Figure 8: it compares four CSI features: mean, standard deviation, skewness and kurtosis, using an ensemble model that implements either FF-NN or LSTM classifiers in each branch $\varphi_{j}$. In particular, the FF-NN consists of one hidden layer of 10 nodes and takes as input either the space or the frequency domain CSI features obtained from T=50 WiFi frames. Likewise, the LSTM network consists of 100 hidden units and extracts sequences of CSI features using a moving window of T=8 consecutive WiFi frames.

As highlighted in the previous sections, the ensemble model learns a classifier (FF-NN or LSTM) on different training sets, namely different CSI features and finally combines the results by voting. Multiple FF-NN and LSTM networks are thus trained using different CSI features and a supervised approach. For both FF-NN and LSTM, 80% of data is used for training, 10% for validation and 10% for testing. The results summarized in Table 3 show that an ensemble model, based on J=11 parallel LSTM classifier networks, provides the best performance with 99% accuracy. Regarding the space-frequency diversity for target counting, the use of space-domain features provides more accurate results (regardless of the deployed classifier, that is, FF-NN or LSTM). For example, an ensemble model that combines J=11 LSTM classifiers and uses statistical average features provides an accuracy of 99% in the space domain. The accuracy drops to 83% when using frequency domain features (and the same ML classifiers). FF-NN classifier networks obtain an accuracy of 95% using space domain features and drops to 81% with frequency domain ones. Therefore, these results confirm that space domain CSI features are more effective in capturing the multipath effects induced by multiple targets. Also, comparing various features used for target counting, the experiments confirm that combining features in both space and frequency domain provides more accurate results.

## 5. Conclusions

In this paper, we proposed device-free target counting using physical layer channel information from a WiFI-MIMO network in the space-frequency domain. Multidimensional statistical channel features are introduced and an ensemble classifier based on machine learning tools is detailed to efficiently use the multidimensional CSI data for target counting. Cloud computing is proposed to collect and classify the channel features in real-time for target counting. Experimental tests conducted to validate the proposed approach performance in indoor environments with crowd size up to N=5 people, give a classification accuracy of about 99% using LSTM and statistical mean feature in the space domain. Future work will focus on two major activities: the exploitation of a physical model to simplify feature extraction during the calibration phase and the introduction of heterogeneous models for ensemble classification.

## Figures and Tables

**Table 1:** Accuracy of subjects counting using maximum likelihood estimation based on normal modelling for the CSI distribution $\mathrm{Pr}[s_{f,\ell ,t}]$ in space and frequency domains.

C	Empty	1	2	3	4	5
space domain	0.44	1	0.84	0.9	0.43	0.55
frequency domain	1	0.9	0.92	0.31	0.54	0.3

**Table 2:** Accuracy of subjects counting using the CSI distribution features and the KL divergence approach in space (10) and frequency domains (9).

C	1	2	3	4	5
space domain	1	0.51	0.43	0.29	0.22
frequency domain	0.93	0.5	0.4	0.39	0.28

**Table 3:** Accuracy of counting subjects using machine learning tools in space and frequency domains.

**FF-NN, Frequency Domain**						
C	Empty	1	2	3	4	5
kurtosis	0.24	0.21	0.68	0.36	0.42	0.557
skewness	0.45	0.956	0.63	0.7	0.59	0.52
standard deviation	0.68	0.78	0.84	0.67	0.58	0.76
mean	0.73	0.9	0.94	0.79	0.67	0.84
**FF-NN, Space Domain**						
C	Empty	1	2	3	4	5
kurtosis	0.31	0.43	0.61	0.46	0.43	0.47
skewness	0.29	0.46	0.54	0.36	0.54	0.62
standard deviation	0.43	0.65	0.89	0.63	0.71	0.78
mean	0.92	0.96	0.98	0.94	0.96	0.92
**LSTM, Frequency Domain**						
C	Empty	1	2	3	4	5
kurtosis	1	1	0.9	0.36	0.63	0.45
skewness	1	0.9	1	0.9	0.27	0.45
standard deviation	0.18	1	1	0.81	0.72	0.9
mean	0.72	0.45	1	0.8	1	1
**LSTM, Space Domain**						
C	Empty	1	2	3	4	5
kurtosis	1	0.9	1	0.63	0.72	0.36
skewness	1	1	1	0.72	0.63	0.54
standard deviation	0.8	1	1	1	1	1
mean	1	1	1	0.9	1	1
